# Extra Supplement—The Nursing Mirror

**Published:** 1891-03-14

**Authors:** 


### "The Hospital" Nursing Mirror.

Being the Extra Nursing Supplement of "The Hospital" Newspaper.

Contributions for this Supplement should be addressed to the Editor, The Hospital, 140, Strand, London, W.C., and should have the word "Nursing" plainly written in left-hand top corner of the envelope.

### En Passant.

SWANSEA INSTITUTE.—The report of last year's nursing work in South Wales is good. Twenty-three private nurses have been at work and one district nurse. Sir John Llewelyn has been elected President, and as Lady Llewelyn is Hon. Treasurer, we foresee for the Institute an even more prosperous career in the future.

WINCHESTER DISTRICT WORK.—The Rev. Canon Humbert presided at the general meeting of subscribers to this charity, which has a Lady Superintendent and one nurse—Miss Prosser. During the year the expenses amounted to £130, and there is a small balance due to the Treasurer. Miss Prosser received much praise for the manner in which she carried out her duties.

KINGSTON NURSING ASSOCIATION.—The annual meeting of this Association was held lately, and a good report was submitted. There are two district nurses, a Lady Superintendent, and a probationer; 293 cases were attended last year. The Committee are considering the desirability of affiliating with the Queen Victoria Jubilee Institute. They also propose to start a district nurse for Surbiton.

BREACH OF PROMISE.—Nurses are women after all, and they occasionally descend to such common-place details as engagements of marriage, hence arises the possibility of breach of promise cases. Mrs. Ada G. M. Watts, a certified private nurse, last week brought such an action against Mr. John C. Mason, and got £175 damages. Mrs. Watts is a widow with two children; in the pursuit of her calling she met the defendant, whose sister she was nursing. He wrote her letters containing rhymes, which, when read in court, caused much amusement. But on second thoughts his admiration for the nurse waned, and he broke off the engagement in sober prose.

FEVER HOSPITALS.—Great interest is being taken in the inquiry into the management of the Eastern Fever Hospital, and once more we must express our admiration of the courage of those who have come forward to express the discontent which has long been smouldering. The fact that the Matron and the doctor go round the wards without putting on wrappers might find a parallel in another London fever hospital where the members of the Committee thus break their own rules. At Birkenhead fresh cases of typhus have occurred, and yet the ratepayers are protesting against the erection of a new hospital, though the present one can never really be fitted to cope with an epidemic. We thought the people of Birkenhead had more pride than to desire another such scandal in their town as the one lately disclosed with regard to their fever hospital. The Belvidere Hospital, Glasgow, was the subject of an inquiry three months ago, and the charges are now being repeated; namely, that the patients are not properly fed, and that the parents are not informed of the serious condition in which small patients may be. With regard to Belvidere and Birkenhead we believe all due improvements have been or will be made, but we would call the attention of the Lords' Committee to the fact that the worst stories of hospital mismanagement occur in those institutions under central control, and that the voluntary supported institutions, into which they are enquiring, come well out of the charges brought against them.

BURTON NURSING INSTITUTION.—A most satisfactory report of this Institution was presented at the annual meeting. There are four day district nurses and one night nurse; Miss Carson is Lady Superintendent. Mr. Worthington, the Hon. Treasurer, is a generous supporter of the Institution, and the Mayor is also interested in the scheme and its workers. The number of cases attended last year was 514.

CHARITY AT CHELTENHAM.—The Charity Organisation Society and the District Nursing Association held their meetings together at the Cheltenham Corn Exchange. The district nurses have lately received a legacy of £1,000, which has enabled them to add a third nurse to their staff. Dr. Wilson, in a brief address, referred to the fact that many visitors who walked up and down the parade, knew little of the poverty of other parts of the town. For eleven years the Charity Organisation Society has done good work in Cheltenham.

SHORT ITEMS.—Mrs. Eliot has been appointed Matron of the Brompton Consumption Hospital.—The series of nursing classes given by Mrs. Franks in aid of the Midwives' Institute were so successful they are to be repeated.—American nurses who nursed through the Civil War are to be exempt from certain taxes.—The North Dublin Guardians have discharged the night-nurse of the Union. It was her duty to visit the Infirmary ward every two hours, but on one occasion she was absent four hours, and during that time two patients died.—The report of the Workhouse Infirmary Nursing Association for March contains severe strictures on the nursing at Richmond Union. - Nurse Britt, of Stanford Union, has been called on to resign for no specified reason, and the Board have granted her a good testimonial. This sounds unfair.—The Jersey Institution issues a good report; 66 cases were nursed last year, 12 on reduced terms, and this, and the work of the district nurse, is the ground on which the Institution solicits subscriptions.

CUMBERLAND INFIRMARY.—On December 17th last the President, the Lord Bishop of the Diocese, brought before the Committee of Management a scheme for grafting on to the Infirmary a means for supplying nurses to private families and for district nursing. The subject was reported on by a sub-committee. On the reception of the report by the Committee, the following amended resolution was passed:—"That the report be received, and if a sum of £2,000 can be raised to meet the initial expenses, and the scheme proposed in the report would make provision for not less than ten private nurses, such scheme should be carried out." The scheme requires the addition of a storey above the out-patients' department, or some such method of obtaining the additional room that would be required. At the late annual meeting, the Bishop again pressed forward the scheme, and said the Mayor had promised a subscription towards it. The present accommodation for the nurses is most inadequate. The new report says:—"The Committee desire to assure the friends of the Infirmary that at no time in its history has the whole institution been in a better state than it is at present. The sick and suffering have never been more skilfully and assiduously attended to. The nursing staff, under Miss Allen, the Matron, are doing an increasingly good work, and carry out the directions of the medical officers with unremitting care."

### The Royal National Pension Fund for Nurses.

The fourth annual general meeting of members of this Fund was held on March 5th ; the following report was adopted :

Pension Branch.—During the twelve months there have been received : 524 proposals (including 33 brought forward from last account) for granting pensions for £8,994 14s. 4d., of which 88 proposals for assuring the sum of £1,435 10s. 1d. are still under consideration, or were not proceeded with ; 436 policies were granted for assuring £13 of immediate annuities ; and £7,546 4s. 3d. of deferred annuities ; producing £4,400 1s. 11d. in single payments, and £4,627 4s. in annual premiums. 1,475 policies have been issued from the commencement of the Fund.

Sickness Branch.—In this department there have been received 154 proposals (including 8 brought forward from last account) for weekly sick-pay assurance of £99 8s., of which 43 proposals for assuring weekly sick-pay of £24 17s. 6d. are still under consideration or were not proceeded with ; 111 policies were granted, securing £74 10s. 6d. weekly sick-pay ; producing £128 8s. in annual premiums. 309 policies have been issued from the commencement of the Fund.

Investments.—The investments on December 31st, 1890, amounted to £73,766 0s. 1d. as follows : In British Government Securities, £20,121 17s. 6d. ; in foreign railway and other debentures and debenture stocks, £29,614 12s. ; in foreign railway shares (preference and ordinary), £5,806 16s. 10d. ; in foreign government securities, £14,455 11s. 3d. ; in municipal corporation bonds and stocks, £3,767 2s. 6d. ; total, £73,766 0s. 1d.

The Council had given long and anxious consideration to the many requests they have received from nurses to be permitted to use the Fund as a savings bank, and to be allowed interest upon premiums withdrawn which had been deposited in the Fund on a returnable scale. It had also been represented that private nurses hesitated to place their savings in the Fund, for fear they might be unable to continue regular payments, and, consequently, after full consideration the Council have determined to grant the following additional privileges to policy-holders on and from January 1st, 1891 : (1) That two-and-a-half per cent. interest be allowed upon returnable premiums withdrawn, less a charge for the expense of administration. (2) That nurses be henceforth permitted to pay into the Fund, irregularly, such amounts as they may be able to deposit with it, which deposits, when they amount to the sum of £10, will be applied to the purchase of a paid-up policy in the Fund on the returnable scale, or to the payment of premiums on existing ordinary policies, as each nurse may prefer.

These concessions are most important, as they give the Fund, so far as nurses are concerned, the advantages of a savings bank, plus the privilege of participation in donation and profit bonuses as they may, from time to time, be declared by the Council.

The Donation Bonus Fund now amounts to about £40,000, a fact upon which the Council congratulate the members and annuitants.

It is gratifying to note the increasing favour with which the Fund is regarded by the leading hospital authorities, and the Council have now prepared a general scheme of federation for the guidance of hospitals and kindred institutions, and there is good reason to hope that no well managed hospital or nursing institution will long remain unfederated to the Fund under a scheme which confers such considerable advantages, not only on the nurse, but also on the federating hospital or institution.

The past year has been an eventful one.

On July 4th the Prince and Princess of Wales received the First Thousand Nurses at Marlborough House, and the Princess presented each of them with a certificate to the effect either : (*a*) that the holder was one of the First Thousand Nurses to join the Fund, and so became one of the founders who secured £25,000 as the nucleus of a permanent trust fund for the benefit of the nurses of the British Empire ; or (*b*) in testimony of her being one of the First Thousand Nurses who joined the Fund unaided by any institution and thereby became entitled to a share of the special bonus fund of £5,000 given by Lord Rothschild, Messrs. Antony Gibbs and Sons, E. A. Hambro, and Junius S. Morgan. No one who was privileged to take part in the proceedings is likely to forget the cordial hospitality of the Prince and Princess on this memorable occasion.

Soon after the reception of the nurses at Marlborough House, July 4th, 1890, Her Majesty the Queen was pleased to command that the Fund should henceforth be called Royal. This spontaneous mark of approval on the part of the Queen cannot fail to add to the usefulness and popularity of the Royal National Pension Fund for Nurses. Her Majesty's recognition is most gratifying to the Council, as it testifies to the Queen's personal sympathy with the objects of the Fund.

On the evening before the reception at Marlborough House, the nurses were entertained at Merchant Taylors' Hall, which was kindly lent for the purpose by the master and wardens, to whom the Council take this opportunity of recording their sense of obligation for the important service thus rendered to the Fund. Every nurse who had joined the Fund was invited, and upwards of twelve hundred were present.

The Council regret extremely to have to report the deaths of Mr. Junius S. Morgan, one of the Vice-Presidents, and of Lady Rosebery, one of the Patronesses. The establishment of the Royal National Pension Fund was largely due to the generous benevolence of the late Mr. Morgan, who gave the first £5,000 to the Pension Fund, subsequently increasing it to £10,000, and whose warm-hearted sympathy with the needs, anxieties, and privations of nurses in the exercise of their hazardous calling has endeared his memory not only to the nurses themselves, but to all who take an interest in their welfare.

From motives of gratitude the nurses determined to raise a memorial to Mr. Morgan, which has taken the form of a Benevolent Fund to be administered by trustees in connection with the Pension Fund. The nurses raised, amongst themselves, no less a sum than £2,268 4s. 7d., which was presented in purses to the Princess of Wales on the occasion of the reception at Marlborough House. On the 18th of December a letter was received from Mr. Walter H. Burns, as the representative of the family of the late Mr. Junius S. Morgan, stating that they were deeply touched by this tribute to his memory, and that his children desired to supplement and increase the amount collected in aid of the Junius S. Morgan Benevolent Fund by raising it to £10,000 in the following proportions, viz. : Mr. J. Pierpont Morgan, £5,000 ; Mrs. Sarah S. Morgan, £1,000 ; Mrs. Mary L. Burns, £1,000 ; Mrs. Juliet P. Morgan, £731 15s. 5d. ; £7,731 15s. 5d.

The Council conveyed their warm thanks to Mr. Burns and the family of the late Mr. Morgan for their munificent gifts, which it was felt would enable the Council to render effectual aid to nurses in the hour of trial and difficulty.

A trust deed has been executed under the seal of the society constituting Messrs. Henry Charles Burdett, Walter Hayes Burns, and Edward Rawlings, and the survivor or survivors of them as the trustees of the Junius S. Morgan Benevolent Fund. The trustees are empowered to invest all sums not required for immediate use, and the income resulting therefrom in any stock, shares, securities, or other investment in which the society may be authorised from time to time to invest its funds.

The trustees will appoint an Advisory Committee, to consist, in addition to the trustees, of such of the (1) patronesses of the society; (2) representative subscribers to the said Fund; (3) annuitants and policy-holders of the Royal National Pension Fund or their representatives (being members of the Council of the Pension Fund), as the trustees may elect, together with the chairman and deputy-chairman of the Council for the time being as *ex-officio* members. The names of the Committee and the objects of the Fund will be found on reference to page 10 of this report.

Only the barest allusion is here made to the important events of the past year, but the Council have arranged to adopt the suggestion of Sir Andrew Clark, M.D., F.R.S., to immediately publish a history of the Fund. Dr. George W. Potter, a warm friend of the movement, has accordingly written a book, entitled, "Ministering Women: a History of the Royal National Pension Fund for Nurses," which will give a full account of the Fund from its foundation to the present time.

COUNCIL AND OFFICERS.—The Countess Cadogan has been elected a Patroness to fill the vacancy caused by the death of the late Countess of Rosebery, and Mr. J. Pierpont Morgan has been elected a Vice-President to fill the vacancy caused by the death of his father, Mr. Junius S. Morgan. In accordance with the Articles of Association, the following five members of the Council retire from office, all of whom being eligible, offer themselves for re-election: Mr. Henry C. Burdett, Mr. E. Murray Ind, Dr. J. S. Bristowe, Mr. P. A. Nairne, and Mr. John Watney. The Articles of Association provide that not more than eight representatives of the Annuitants and Policy-holders of the Society may be added to the Council. The Council have, therefore, nominated the following five ladies for election by the Annuitants and Policy-holders: Miss L. M. Gordon, St. Thomas's Hospital; Miss E. Vincent, Lady Superintendent, St. Marylebone Infirmary; Miss M. L. Waters, Assistant Matron, the London Hospital; Miss A. Ross, Victoria Infirmary, Glasgow; Miss S. Hale, the Hospital, Rotherham, Yorkshire.

We hope next week to give the speech of Mr. Walter H. Burns in which he moved the adoption of this report. Meanwhile, we have only room to congratulate the members of the Pension Fund on the success of the movement.

### Princess Christian's Daughter.

MISS E. DURHAM, Farringford, Freshwater, Isle of Wight, acknowledges the following additional subscriptions towards a wedding present for Princess Louise of Schleswig-Holstein to be given as a proof of the gratitude of nurses for the interest Princess Christian has ever taken in their progress. Subscriptions will be received until the end of March, and will be acknowledged in these pages. Between March 2nd and March 9th the following sums were received:—

Sister Marr, 2s. 6d. H. Foggo Thomson, 2s. *Nurses* (1s. each): Charge Nurse M. A. Chaplin, Charge Nurse Amy Campbell, Charge Nurse Alberta M. Webb, Charge Nurse Harriet Jordan, L. Hart, S. A. Baker, Ethel Strachan, B.N.A., E. M. Quinn, A. H. Fielder, M. J. Grey, S. C. Bothams, Ada, Rodgate, Emma Wisby, George, Adkins, Featon, Mackaness, and Wilcox.

THE set of classes on "Home Nursing," which were so kindly given by Mrs. Franks in aid of the funds of the Midwives' Institute, were so successful that they will be shortly repeated in aid of the Haggerston District Nursing. Anyone wishing to attend can obtain particulars by sending a stamped and directed envelope to Mrs. Nichol, Nurses' Club, 12, Buckingham Street, Strand.

### The Eastern Hospital Scandal.

MR. HEDLEY and Dr. Bridges opened the inquiry into the administration of the Eastern Hospitals on March 3rd. Mr. Simpkin, the first witness, said he was a patient from June 30th to September 3rd last year. The food was bad, and not in accordance with the dietary scale. He frequently complained—once to Dr. Collie, and on other occasions to the nurse. When he complained to the doctor he had a piece of haddock before him. Dr. Collie smelt it, and said he could detect nothing wrong. Witness asked him to taste it, which he did, and said it was very nice. In the opinion of the witness the fish was nearly rotten. His portion was afterwards taken away, and he had two chops for his meal instead. Generally speaking, the dinners were supplied either very cold or very warm. They only had mutton. Mr. Hedley: Always mutton? Witness: Yes; always mutton. There was no tooth brushes in that ward, but there was one in the Truth Ward, which was used for the ward generally. It was also used to clean children's tongues with Condy's fluid. There were only about half a dozen brushes and combs in the wards; they were in bad condition. Poultices were mixed in the same mugs as those from which the patients had their tea. He saw on several occasions four and five children in the bath at once. One of the children was recovering from chicken-pox, while the others were suffering from scarlet fever. In the mornings several children in the acute stages were washed in the same basin. The next witness, Miss Diane Halkin, deposed that at present she was a temporary staff nurse at St. Thomas's Hospital. She was charge nurse at the Eastern Hospital for over four years. While Simpkin was in the Truth ward she was night nurse there. Speaking generally, the character of the food supplied to the patients while Simpkin was a patient was inferior. It had been very bad for a long time, and got worse at the time Simpkin was there. On one occasion she broke a loaf in two, and it was mouldy inside. She got it changed, and the second loaf was mouse eaten. This was in 1888. Cheese was never supplied for the patients during the whole time she was charge nurse, and she never saw cocoa supplied, although both these items were on the dietary scale. It would be quite true to say that the fish was unfit for human food. She was day nurse for over three years, and the dinners always consisted of boiled mutton, which was very fat, very inferior, sometimes tainted, mixed with bone, and deficient in quantity. The milk supplied was often sour, and the quantity nearly always short. She had complained to Dr. Collie about various matters, and had drawn his attention to the bad quality of the food supplied on several occasions. She said she had seen visitors in a room where a diphtheria patient was being bathed. In the same room some of the child patients had their hair cut, and she had seen the dresses of the visitors sweeping this hair along the floor. She had seen Dr. Collie, in Mare Street, Hackney, in the same clothes as he wore in the wards, and she had seen the Matron go out in the same dress she had just before worn in going round the wards. She had seen both Dr. Collie and the Matron going round the diphtheria and scarlet fever wards without being provided with wrappers. She had seen other officials besides the Matron going round the wards in their outdoor clothing. In cross-examination, Nurse Halkin was asked how she got her present appointment at St. Thomas's, and if she ever gave children money, or abstracted fish or cream intended for the patients. Nurse Halkin had ready answers for all insinuations.

Mr. Benson, postman, and Mr. Oldfield, printer, both of whom were patients in the hospital last autumn, stigmatised the food as bad, and not in accordance with the diet scale. Mr. Jay, clerk, said the food was disgraceful, and otherwise corroborated the evidence of previous witnesses. The inquiry was proceeding when we went to press.

### A Night's Experience at Kimberley Hospital.

Kimberley Hospital, which can accommodate 317 sick, consists of an irregular group of buildings, scattered over a a wide, partially enclosed space. The main building is a large brick bungalow, surrounded by broad verandahs, or stoeps, as they are called here; and containing the European wards, male, female, and children's, in all 70 beds; the operation-room, nurses' dining-room, European kitchen and offices; also a few bed-rooms, in which some of the nurses sleep. All the rooms are on the ground floor, and open on the stoeps. Behind this block is the compound in which are built the native wards, each a separate block, one might almost say shed. These are six in number. The male native surgical, the show ward of the whole hospital, beautifully kept, admirably worked, and with its own operation-room attached to it; the male native medical; two native female wards; a block containing European and native lock wards; and a large convalescent ward in which there are no beds, the natives sleeping on the floor swathed in blankets, faces and heads covered, in a fashion that gives them a strange and mummy-like appearance.

Besides these, the compound contains isolation wards, native kitchens, dispensary, houses of resident doctors, chapel, mortuary, etc.; also sheds in which the native servants sleep. Each ward has its night-boy and day-boys, and these native servants, whether ward, kitchen, or laundry boys, are the plagues of the hospital. Idle, noisy, tipsy, dishonest, they bring with them an element of noise and disorder with which no organisation seems able to cope.

Every now and then the more respectable among them disappear for 24 hours, retiring to the Malay camp, in the neighbourhood of the hospital, a paradise of noise, whose inhabitants beat the tom tom and yell for eight or ten hours at a stretch, and where drinking, fighting, and haschisch smoking are always going on. The others are less discreet, and the nurses have learnt to dread the arrival of the monthly pay-day, which is invariably followed by scenes of drunken riot and confusion.

Coming on duty on the evening of January 5th, I find that only two night-boys are sober, and that almost all the day-boys are drunk. As I make my first round, and cross the compound towards the isolation wards, I see the "boys" (so the natives are styled, even when they are ninety) lying on the ground outside their shed, rolling about, laughing noisily, but, so far, not inclined to be quarrelsome. The night wears peacefully away till nearly 1 a.m., when suddenly a great clamour arises in the compound; I hear a confused sound of laughter, shouts, and groans. I look for the policeman who patrols the hospital grounds at night, but the police all over the world seem to possess a mysterious power of becoming invisible the moment they are wanted, and our guardian genius is nowhere to be found. I hurry up to where the natives are assembled, and in a strange jargon of English and a Kaffir word or two, I urge them to be quiet and not disturb the patients. They say "Yes, missus," good-naturedly enough, and for a moment are quiet. I return to the European kitchen, and have just seen the last of the night-nurses' supper-trays carried off, when a man rushes in and tells me that the boys are fighting, and that one of them is being killed. At the same moment a perfect storm of yells breaks out.

I catch sight of the policeman, who apparently hears and sees nothing, for he seems quite amazed when I tell him that a disturbance is going on, and that he must go and separate the combatants. However, he starts off willingly enough, and yet, being an Irishman, I should have thought he'd have been more eager to join the fray. He goes, and a pause ensues, when suddenly a powerful and infuriated Kaffir dashes up to the kitchen shouting for a knife. He darts into the scullery, the policeman rushes in after him and drags him out; they struggle fiercely. The brass handle of a tap is snapped off as if it were a reed; a water-pipe is torn down and smashed; the water pours into the compound, and out into it, rolling over and over, go policeman and Kaffir. The native is very strong, and it seems impossible to get the handcuffs on. Several times he shakes the policeman off and makes for the kitchen and the knives. Other natives rush up; the two ward-boys who are sober appear, but refuse to help. At last, three or four men who are going round with the sanitary carts come to the rescue, and the fight becomes general. The lovely starlit southern night is made hideous with shrieks and oaths. All this occurs under the open windows of one of the wards; the sick people awake, terrified; a typhoid gets out of bed; it is all the night-nurse can do to keep order inside. I slip away, run to the house of the Senior H.S., and, knocking at his window, tell him what is happening. In less than five minutes he is at the scene of action. By this time the Kaffir is handcuffed, but is still violent and refuses to go. Wet through, covered with mud from head to foot, the policeman is a pitiable object, looks rather helpless, and one doesn't feel sure whether he is not his prisoner's prisoner.

The H.S., Dr. Callender, soon settles the matter. Seizing the handcuffs he drags the Kaffir up from the ground, and marches him down towards the gate, where he gives him up to the policeman, who now finds no difficulty in leading him away. As they dissappear Dr. Callender strides off to the Kaffir quarters, and I follow, for I have been told that riotous natives are often less violent if a nurse is present. On the way we meet four big natives, who are very drunk, and who appear inclined to be insolent. The H.S. pounces on two of them, shakes them as a terrier shakes a rat, and drives all four into the shed. This is crowded with natives, all more or less drunk and very riotous. There is a strong smell of cango (African brandy), and the place is lit up by a number of candle ends stuck in bottles. We had found one huge native in a sort of out-house; he was just tipsy enough to be dangerous, and had obeyed orders very sullenly, muttering threats as he reeled up to the shed. We find him established on an old bedstead brandishing a bottle as if he meant mischief. I am horrified to see the H.S. walk into the shed, and order the lights to be put out; at first no one stirs, and one or two give a derisive yell, but at last they obey, and we turn to go. Suddenly the man on the bed strikes a match, relights his candle, and tells Dr. Callender to get out, and "get out or we'll do for you," shriek all the others in chorus. "You tell me to get out? Wait a little my friends!" and the H.S. runs along the compound and blows his fire whistle; he then darts to the place where the fire-hose is kept. In obedience to the whistle a white man appears, and in three minutes by my watch from the time the whistle blew, the hose is ready to function. By this time the native shed is again illuminated—it is a marvel that it hasn't long since caught fire—dancing is going on in it, and shouts of laughter are heard—evidently at our expense.

As the H.S. again appears at the door of the shed a general shout of "Get out" is raised. Foremost among the shouters is the big man on the bed, who lifts his bottle threateningly. As he does so the hose is turned on him, the water dashes into his mouth—in a second he is wet from head to foot, almost sober and perfectly quiet. I never see him move again.

A scene of the wildest confusion follows, the boys rush madly about the shed trying to escape from the hose which finds them out in every corner of the place. They try and make a dash at the door, but the water drives them back.

At last, very much sobered, they throw themselves on the ground; the lights are out, and, swamped as it is, no one dares to leave the shed till morning, when a dismal procession of very meek-looking boys emerges from it, carrying wet shirts and blankets to dry in the sun.

Meanwhile Dr. Callender plugs the broken water pipes, restores things in general to something like order, and departs to get into dry clothes, promising not to go to bed, but to go round at intervals till morning. He has, however, no further trouble, the Kaffirs being thoroughly cowed.

This trouble with the natives, though happily, as a rule, in a milder form, occurs after every pay-day. It seems a pity; for to find such a hospital as this, with its Training School for Nurses and its beautifully-kept Nurses' Home, in the wilds of Africa, is truly astonishing and delightful. It is an institution of which Kimberley may well be proud, but it is at all times an extremely noisy place, often intolerably noisy, as during the night I have been describing. There being no drains in Kimberley, ward-boys are indispensable to carry away slops, fetch water, &c. Necessarily these boys are all over the place, and clatter follows in their wake. Some say that if the hospital authorities could afford to pay better wages they could secure the services of a better class of boys. Others say that the boys are all alike, and that no improvement can be hoped for till the natives fall under the influence of advancing civilization. No doubt they will then give up brawling and cango in favour of tea and cheap literature, and the penny dreadful and shilling shocker will not have existed in vain.

Meanwhile, I think that whoever aspires to the post of H.S. at Kimberley Hospital should not only be doubly qualified, but have a certificate for presence of mind and pluck. Rose Blennerhassett.

### The Nurses' Bookshelf.

### ABOUT BABIES.*

"Babyhood" calls itself a magazine for mothers, but we are sure it ought to be of the utmost use to nurses as well. Not only are there excellent articles by medical men on such subjects as "Tuberculosis," "Rickets," and "Nursing in Diseases of Children," but the queries asked and answered just touch on those points which most books ignore. We note that some of the answers are sent by trained nurses, so that evidently our American sisters have learnt to appreciate this magazine. The anecdotes about children are very amusing and very instructive; most of them have a Yankee flavour, and refer to remarkable "'cute' babies. Still, it is very nice to come across a volume which is never dull, though crammed full of instruction.

Mrs. Hewer must be known to many of our readers as the author of the "Manual on Antiseptics," and they will gladly welcome a new work from her pen. "Our Baby" apparently arouses a lot of difficulties from the moment of its arrival, and what to do with the infant, how to clothe and feed it and nurse it through its illnesses, are the points dealt with by Mrs. Hewer. There is nothing amusing in this volume, it is a text-book to be conned seriously and turned to in trouble. It has the great advantage of being neither too long nor too short, and it contains an exhaustive index. According to the preface, "this little book has been written for those mothers and nurses who know that there must be a right way of bringing up a child, and who are anxious to know what that right way is." And the book fulfils its purpose.

Mrs. Bowditch, in her "Chats with Mothers," deals chiefly with everyday matters and does not venture on the subject of illnesses. Her remarks are very sensible; she has great faith in nature, and speaks severely of those who would rouse sleeping children or cram them with food when they are not hungry, or prevent them from sucking their thumbs. Is it possible that animals can rear their young, and yet human mothers need all this instruction about trifles?

* "Babyhood": A Monthly Magazine for Mothers. London: (Babyhood Publishing Offices, 92, Fleet Street.) Price 6d. "Our Baby." By Mrs. Langton Hewer. (Bristol: Messrs. Wright and Co.) Price 1s. 6d. "Confidential Chats with Mothers." By Mrs. Bowditch. (London: Messrs. Bailliere, Tindall, and Cox.)

### Everybody's Opinion.

### PADDINGTON GREEN CHILDREN'S HOSPITAL.

Mr. W. H. Pearce, the Secretary, writes under date March 9th: I am directed by the Committee of the Paddington Green Children's Hospital to inform you that they have thoroughly investigated the charges conveyed by Mrs. Phillips' letter in your paper of the 24th January last. Briefly, the facts are the following: (1st) All clothes, blankets as well as sheets, were removed from the bed occupied by the first nurse who left the hospital ill, and Nurse Bishop's bed was made with clean clothes by Nurse Bishop herself, with the assistance of one of the maids. (2nd) On the morning of the 15th November (Nurse Bishop having fainted on the night of the 14th) the Matron asked her to see one of the medical staff; but believing that one of her "old attacks" (Nurse Bishop had previously suffered from some obscure complaint, which she herself said was regarded as tubercular) was coming on, she said she would go home at once and place herself under the care of her own doctor. To the Matron and her fellow nurses, all of whom have been carefully questioned, Nurse Bishop presented no signs of such weakness as the journey to Tooting seems to have brought out, and no doubt arose in the Matron's mind as to Nurse Bishop's ability to go home alone. As to the alleged high temperatures which Mrs. Phillips reports on the Tuesday and Wednesday before Nurse Bishop left the hospital, no one in the hospital knew even that Nurse Bishop was taking her temperature. Nurse Bishop's temperature was taken on the night of the 14th and morning of 15th November, and was found to be normal. (3rd) With regard to Nurse Phillips, she was given a month's notice on November 22nd, and accordingly left on December 22nd, being at that time, so far as her fellow-nurses and the Matron knew, perfectly well. The nurses have been questioned, and neither the nurse who shared a room with Nurse Phillips, nor the nurse on duty with her, knew either that she was ill, or that she was suffering from diarrhœa, as alleged. (4th) With respect to the sanitary system of the hospital, an immediate investigation was ordered, and has now been carried out, the result being that no serious defect has been discovered by the surveyors. Some minor alterations were suggested, and are now receiving attention. (5th) In conclusion, the Committee are satisfied that no blame whatever attaches to the Matron or other official of the hospital in the above matters. The insertion of this in your next issue will be esteemed by my Committee.

*** We would point out that—(1) The bed, as well as the bedding, ought to have been removed entirely, and the room disinfected. (2) This shows the importance of the strict enforcement of a rule to the effect that every nurse on the staff, when unwell, shall be at once visited by the medical officer to the nurses. We should like to have a little more information as to the character of "the minor alterations in the sanitary system of the hospital," to which reference is made.—Ed. *T. H.*

### PERSONAL ANECDOTES.

Sister Eva writes: "Allow me to correct an error with reference to your notice of my little book ("Scenes in the Life of a Nurse"). The price is one shilling; only a limited number of the two shilling edition have been prepared, and they are only supplied on request.

### Presentations.

On February 27th the nurses of the City of Dublin Institution presented to Mr. William Ireland Wheeler the following address, beautifully illuminated: "We, the undersigned, wish to express our deep regret at your retirement from the post of Honorary Secretary to this institution. We also desire to record our gratitude for the impartial, kindly care, and energy ever bestowed by you upon all that concerned our welfare, as well as for the independence which you, as the originator of the institution, have placed within the reach of all of us We beg your acceptance of our earnest wishes for your long life, happiness, and ever-increasing success." Mr. Wheeler was instrumental in founding the institution, and has ever been its firm friend.

Broomhill Home for Incurables.—On February 27th the nurses and servants assembled in the board-room and presented to Miss Winder, the retiring Matron, a handsome gold bracelet set with pearls; and to Miss Kay, Head Nurse, who has got another appointment, a beautiful gold brooch. Dr. Whitelaw replied for the ladies.

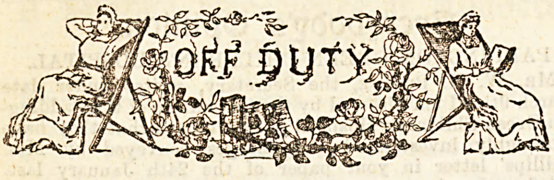

### Rumples.

(Addressed to Our Country Cousins.)

She sat on the edge of her narrow bed, sewing away for dear life, a pale-faced nurse, with dark circles under her eyes. Around the opening of the cubicle were several other nurses in various stages of undressing; and throughout the big dormitory appertaining to the pros. arose the mingled voices and rustlings that betoken the near approach of 10.30 p.m.

"I wish the doctor had ordered me a holiday—aren't you glad to get away, Jessie?" said one of the scantily-clothed damsels, eyeing covetously the half-filled trunk on the floor.

"Well," said the owner of the trunk, as she snapped off another piece of cotton, "I am not very sure that I am glad. You see," she went on, half in fun and half in earnest, "I live in a district planted out with relations, and they won't let me have one day to myself. Aunts and cousins come to call on me in shoals, and of course I must return their visits and stay to tea with each of them in turn—if I didn't they would never speak to me again. I should not mind that, but mother would. But the tea is not the worst, for all the cousins have a special recipe for black currant wine—I hate currants in any form—and if you do not taste it they are so mortified. It is of not the slightest use to say you belong to a Band of Hope, for home-made wine, according to the cousins, is the favourite beverage of abstainers, and quite unintoxicating. Then the cakes, tarts, and pies, that are made for my special benefit, and of which I am obliged to partake, would stock a dozen confectioners. It does not mend matters to say you are on invalid diet, for straightway every aunt prepares a different kind of herb tea, and all the other relatives, up to the tenth degree, make soups and jellies and broths, and they drive over with basins and jugs, and administer the contents to you with a kindly but firm hand. Then they stay the rest of the day to cheer you up. They recall to your mind all the little tales of your youthful days, which you had hoped were forgotten, and won't go home till midnight, because the moon doesn't rise till then. All the while you are wishing you lived in a moated grange, with the drawbridge in good working order. Then, you know, they don't take kindly to uniform in agricultural districts, excepting the small boys, who hail you with shouts, and follow you about as they would Sequah or Buffalo Bill. An entirely new rig-out is more than a poor pro. can afford, and so in the end one buys a cheap gown, and trims up an old hat, and has the satisfaction of looking the shabbiest cousin of the crowd. The country girls have such dainty cambrics and prints, while mine—well, you know how they look after a year's hospital washing. I have nearly stitched myself to death the last few days."

Just then the rustle of a gown was heard in the corridor, and each nurse, dressed and undressed, made for the nearest bed. The gas went out with a jerk, and a second later, when the Lady Superintendent looked in, the pros. were sleeping with all their might and main, their deep, regular breathing speaking well for their lung power. From one bed, indeed, the sounds came somewhat in gusts, but that was because the three pros. who had scrambled in together were trying to breathe in turns, and naturally failing. I am sorry to say, also, that a resonant nasal sound issued from one corner, but that cubicle was occupied by a new-comer who had not yet acquired that propriety of demeanour which we all recognize as the distinctive characteristic of the pro.

The Lady Superintendent smiled and went her way. A few minutes later the voices and rustlings commenced again as the nurses sorted themselves, each to her own bed. But Jessie was fast asleep, more tired with her enforced preparations than by a hard day's ward work.

### Notes and Queries.

### Queries.

*Public Health.*—What are the names and prices of journals devoted entirely to public health?—D. P. H.

### Answers.

*Royal National Pension Fund.*—Nurse Simpson's letter has been forwarded to the Manager of the Fund (8, King Street, Cheapside, E.C.).

(42) Apply to the Matron, Melicent Home, Sandown, Isle of Wight. Incurable children are taken there; consumptive cases, 7s. 6d. a week; surgical cases, 8s. a week.

*C. N. H.*—Robin's ready-made linseed poultice, patented by Messrs. Sealbury and Johnson.

(34) Pneumonia jacket. Take a piece of flannel sufficient to go round patient; cut two half-moons at top for arm-holes; stitch two strips of flannel to the top at back. Pin the jacket in front, bring the straps over the shoulders and pin them in front; this will keep the poultice on, and you can change it without difficulty.—*L. Watson Tulloh.* [We hope to publish shortly the illustration sent with this answer.]

*Wren.*—It is a standing rule, to which we can make no exception, that the names of particular practitioners are never given. You should consult your own medical man about any specialist you may desire to see.

*M. H.*—See answer to *Wren* above.

*An Old Nurse and Midwife.*—We agree with your letter, but think it wiser not to publish it. You can safely leave your cause in the hands of the Doctor who occupies the Armchair.

*H. H.*—We really do not see why nurses should send their payments to the Pension Fund post free. We think they have enough privileges already.

(35) *Nursing in New York.*—It is very difficult for an English trained nurse to get a post in a New York hospital, though English nurses can get plenty of private practice here.—*T.*

(36) *Carbolic.*—The general opinion is that carbolic is the best disinfectant in scarlet fever, but full particulars can be had in "Antiseptics," by Mrs. Hewer, published by Crosby Lockwood. Price 1s.

(37) *Hydropathics.*—The usual charge is from two to four guineas a week. The cheapest I was ever at was the Deeside, near Aberdeen, where, by sharing a bedroom, terms are reduced below those given above. It is best to take the advice of a medical man as to what hydropathic to go to.—*Scot.*

(40) Most hospitals admit on their staff nurses not of their own training, for instance Royal Hants Infirmary, Great Ormond Street Hospital for Children, Marylebone Infirmary, the Seamen's Hospital, Greenwich, and many others.

(41) *Edith* had better call at this office (140, Strand) and see the nurse dolls and copy the cap she wants.

*Veri as.*—Please send name and address.

(42.) If "Incurable" will write to Miss Walmsley, Home for Incurables, Upper Parliament Street, Liverpool, the desired information will be forwarded.

*Mina.*—We give a letter from abroad every week—either France, Buenos Ayres, Johannesburg, New York, Melbourne, or some town where trained nursing is carried on. We must refer you to these in back numbers. We cannot advise you where to go so long as your desires are so vague.

### Appointment.

Bristol General Hospital.—Miss Mary Eaton has been appointed Assistant Matron (not Matron, as stated last week) to this hospital.

### Amusements and Relaxation.

### SPECIAL NOTICE TO CORRESPONDENTS.

First quarterly word competition commenced January 3rd, 1891; ends March 28th, 1891.

Competitors can enter for *all* quarterly competitions, but no competitor can take more than *one first prize* or *two* prizes of any kind during the year.

Proper names, abbreviations, foreign words, words of less than four letters, and repetitions are barred; plurals, and past and present participles of verbs, are allowed. Nuttall's *Standard* dictionary only to be used.

N.B.—Word dissections must be sent in WEEKLY not later than the first post on Thursday to the *Prize Editor*, 140, Strand, W.C., arranged alphabetically, with correct total affixed.

The word for dissection for this, the ELEVENTH week of the quarter, being "ANEMONES."

| Names. | March 5th. | Totals. | Names. | March 5th. | Totals. |
|---|---|---|---|---|---|
| Reynard | — | 77 | Woodbine | — | 25 |
| Reldas | 106 | 520 | Madame B. | — | 25 |
| Tinie | — | 30 | Shakespeare | — | 59 |
| Patience | — | 76 | Smyrna | — | 259 |
| Jenny Wren | 96 | 464 | Southwood | — | 102 |
| Agamemnon | 106 | 513 | Gipsy Queen | — | 21 |
| Wyameris | — | — | Snowball | — | 19 |
| E. C. | 104 | 510 | Rita | — | 88 |
| Ecila | — | — | Mortal | — | 16 |
| Hope | 106 | 517 | Nurse Annie | — | 15 |
| M. W. | 104 | 506 | Carmen | — | 11 |
| Qu'appelle | 107 | 502 | Grannie | — | 45 |
| Nil Desperandum | 101 | 510 | Amie | — | 30 |
| Lady Betty | 88 | 473 | M. R. | — | 25 |
| H. A. S. | 75 | 416 | Primrose | — | 24 |
| Sister Jack | — | 62 | Nurse J. S. | 81 | 280 |
| Crystal | — | 203 | B. A. C. | 85 | 209 |
| | | | Theta | 84 | 105 |

